# Characterization of *Salvia Miltiorrhiza *ethanol extract as an anti-osteoporotic agent

**DOI:** 10.1186/1472-6882-11-120

**Published:** 2011-11-28

**Authors:** Yan Cui, Bidur Bhandary, Anu Marahatta, Geum-Hwa Lee, Bo Li, Do-Sung Kim, Soo-Wan Chae, Hyung-Ryong Kim, Han-Jung Chae

**Affiliations:** 1 Department of Pharmacology and Institute of Cardiovascular Research, School of Medicine, Chonbuk National University, Jeonju, Chonbuk, South Korea; 2 Department of Dental Pharmacology and Wonkwang Dental Research Institute, School of Dentistry, Wonkwang University, Iksan, Chonbuk, South Korea

**Keywords:** *Salvia miltiorrhiza*, OVX, BMD, morphometry, oxidative stress

## Abstract

**Background:**

*Salvia miltiorrhiza *(SM) has long been used as a traditional oriental medicine for cardiovascular disease. Accumulating evidence also indicates that SM has anti-osteoporotic effects. This study was conducted to examine the SM-induced anti-osteoporotic effect and its possible mechanisms with various doses of SM.

**Methods:**

We studied Sprague-Dawley female rats aged 12 weeks, divided into six groups: sham-operated control (SHAM), OVX rats supplemented with SM (1, 3, 10 and 30 mg/kg) orally for 8 weeks. At the end of the experiment, blood samples were collected and biochemistry analysis was performed. Specimens from both tibia and liver were processed for light microscopic examination. DEXA and μ-CT analyses of the tibia were also performed.

**Results:**

SM treatment significantly ameliorated the decrease in BMD and trabecular bone mass according to DEXA and trabecular bone architecture analysis of trabecular bone structural parameters by μ-CT scanning. In serum biochemical analysis, SM decreased the released TRAP-5b, an osteoclast activation marker and oxidative stress parameters including MDA and NO induced by OVX.

**Conclusions:**

The preventive effect of SM was presumably due to its anti-oxidative stress partly via modulation of osteoclast maturation and number. In current study, SM appears to be a promising osteoporosis therapeutic natural product.

## Backgrounds

Osteoporosis is a multifactorial skeletal disease that is characterized by compromised bone strength predisposing a person to an increased risk of fracture [1]. Long-term administration of currently prevalent medications may lead to an increased risk of severe side effects like cancer [2]. *Salvia miltiorrhiza *(SM) has long been used as a traditional oriental medicine for cardiovascular disease. Accumulating evidence also indicates that SM has anti-osteoporotic effects. The dried root of *Salvia miltiorrhiza *Bunge (Labiatae) is also named Danshen or Tanshen. The herb is mainly produced in Anhui, Shanxi, Hebei, Shuan, and Jiangsu provinces in China [3]. Among the chemical constituents of Danshen, there are tanshinone I, tanshinone IIA, tanshinone IIB, cryptotanshinone, tanshindiol C, 15,16-dihydrotanshinone I, isotanshinone I, isotanshinone II and other tanshinones [4]. Several biological activities have been detected for the major tanshinones through *in vivo *and/or *in vitro *tests, such as antioxidant, anti-inflammatory, antimicrobial, anti-menopausal syndrome, anti-ischemic, and antineoplastic activities [3,5,6]. The inhibitory effect of tanshinone IIA on osteoclast differentiation and bone resorption was also observed [7]. Consistently, SM significantly increases the blood estrogen level in ovariectomized (OVX) rats, suggesting that SM might help prevent bone resorption in this osteoporosis model [7,8]. These results were also related with a study suggesting that SM has a positive effect on promoting angiogenesis [9]. Wong et al. also showed that SM extract increased bone formation through the combined actions of increasing angiogenesis, increasing osteoblastic activity and decreasing osteoclastic activity [10,11]. Our previous study revealed that aqueous extract of SM effectively prevents the development of bone loss induced by OVX in rats [12]. However, a detailed characterization of the effect of SM has not been elucidated yet.

The aim of the current study is to clarify the anti-osteoporotic effect of SM at various doses. This study was performed in OVX rats by observing the changes in biochemistry data, bone mineral density (BMD), trabecular bone structural morphometric traits and histological characteristics.

## Methods

### Materials

The dried root slices of SM were acquired from Hansol Oriental Medical (Gimje, Korea). 1800 g of SM powder were prepared from dried root slices of SM that were cut into small pieces and extracted with 100% ethanol at 78°C for 3 hr in triplicate. The extract was filtered, evaporated on a rotary vacuum evaporator at 50°C and freeze-dried to yield 26.52 g of SM extract. 106.56 μg of tanshinone IIA/10 mg of SM extracts (1.07%) and 109.655 μg of crytotanshinone/10 mg of SM extracts (1.10%) was verified by high performance liquid chromatography (HPLC).

The chemical products used in the experiment include: methanol and acetic acid of HPLC grade (Merck, Germany). Tanshinone IIA and cryptotanshinone standards were purchased from Sigma Company (USA). Rompun (1 ml of Rompun contains 23.32 mg of Xylazine hydrochloride) was purchased from Bayer Korea (Ansan, Korea) and Ketamine was acquired from Yuhan (Seoul, Korea). Estradiol Depot was obtained from Jenapharm (KG, Germany).

### Experimental Animals

Twelve-week-old female Sprague-Dawley rats, weighing 230-270 g, were purchased from Damul Science Co (Daejeon, Korea), allowed to acclimate for 7 days, and kept another 7 days for a baseline period before the start of the experiment. The rats were maintained at a constant temperature (25 ± 2°C) and humidity (55% ± 5%), with a cycle of 12 hours light and 12 hours darkness. They were housed individually in standard cages and were provided with *ad libitum *tap water and a commercial standard diet containing 1.2% calcium and 0.8% phosphorus. All procedures using animals were carried out in accordance with the guidelines presented in the "Principles for the Care and Use of Animals in the Field of Physiological Sciences", published by the Physiological Society of Korea. The study protocol was approved by an ethics committee in Chonbuk National University (Jeonju, Korea). Experiment animals were allocated to sham-operated (Sham), OVX-control (OVX), and 1, 3, 10 and 30 mg/kg SM treated (1SM, 3SM, 10SM and 30SM) ovariectomized groups for a total of 6 groups (N = 10/each group). Rats in the sham-operated group underwent a sham operation, i.e., only the skin incision was made. Briefly, the operations were performed by exteriorizing the ovaries after the baseline period at week 0; the other rats were ovariectomized. Rats were operated on while under anesthesia by a mixture of Ketamine (35 mg/kg) and Xylazine (10 mg/kg) administrated intraperitoneally. Success of OVX was confirmed at necropsy by retrospectively inspecting atrophy of the uterine horns [13,14]. After a 1-week healing period, rats in the drug-treated ovariectomized groups were orally treated with a series dosage of SM once daily for 8 weeks and Sham and OVX groups were orally treated with volume-matched vehicles before sampling. The doses and durations of SM treatment were predetermined on the basis of preliminary studies [15]. The body weight of each rat was measured weekly, and the dosage of drug or vehicle administered was calculated based on the most recent body weight measurement. After 8 weeks of drug administration, the experimental rats were fasted overnight; the next morning, rats were anesthetized and blood was sampled from the abdominal aorta. Serum was isolated from the blood samples by centrifugation at 3000 × g, 4°C, for 5 min and stored at -70°C prior to biochemical measurement. After the blood sample was collected, the rats were bled to death, and the liver and tibiae were excised. The liver and left tibia (defleshed) of each animal were fixed with fixative and used for further histomorphometric analysis, while the right tibia was freed of all soft tissue and wrapped in a layer of PARAFILM (Menasha co., U.S.A), apart from 5 mm of its proximal end, and fixed into a 15 ml BD Falcon Tube (BD Co., Franklin Lakes, NJ USA) and then soaked in fixative. The tube cap was tightened before performing a μ-CT scan to measure the microstructural parameters. The right femurs were subjected to DEXA measurement for BMD and bone mineral content (BMC).

### Bone μ-CT Scanning

To assess bone loss, rats right tibiae (defleshed) were *ex vivo *scanned at the end of drug treatment. A 6-mm μ-CT scan (70 kV, 85 lA, 1,000 projections per 180 degrees, 350 ms integration time) with an isotropic resolution of 18 μm was made of the proximal tibia using an *in vivo *μ-CT scanner (SkyScan-1076 *in vivo *CT-scanner; SkyScan, Belgium) The CT scanner was calibrated, and a beam hardening correction algorithm was applied to all scans. One CT scan took 35 minutes. In this study, the reproducibility of all structural parameters was high, with a coefficient of variation of about 1%.

From the stack of cross-section images, a volume of interest (VOI) containing only cancellous bone was extracted for morphometric analysis (CT Analyzer V 1.11.0.0, Skyscan, Kontich, Belgium). The VOI started at a distance of 1 mm from the lower end of the growth plate and extended distally for 110 cross sections (2 mm in height). For morphometric analysis, the following structural parameters were calculated over each VOI of cancellous bone by "3D analysis" (using CT Analyzer software): bone volume fraction (BV/TV), connectivity density (Conn.D), trabecular thickness (Tb.Th), direct trabecular separation (Tb.Sp), trabecular number (Tb.N), trabecular pattern factor (Tb.Pf), BMD, and structure model index (SMI). SMI indicates whether the trabeculae are more rod-like (SMI = 3) or more plate-like (SMI = 0); Lower Tb.Pf signifies better connected trabecular lattices while higher Tb.Pf means a more disconnected trabecular structure; Conn.D was obtained by calculating the connectivity of the trabecular network and normalized by dividing the connectivity by bone volume (BV/TV) [14].

The cortical area of the diaphyseal region of the tibia was also calculated using CT Analyzer software. The cut level for measurement of the cortical area was defined at a distance of 8 mm from the lower end of the growth plate. The cortical area (Ct.Ar), and cortical thickness (Ct.Th) were analyzed by "Individual 2D object analysis" in CT Analyzer software, and cortical thickness was calculated by the formula Ct.Th = 1/2 × BS/BV. The above formula is defined as: area of a ring = thickness of ring × length of middle line = thickness × (outer circumference + inner circumference)/2 [14].

The average attenuation coefficient of the trabecular bone tissue was determined for all measurements using a protocol provided by the manufacturer of the μ-CT scanner. With this protocol, the gray levels of voxels near the trabecular surfaces are not included to ensure that the measurements are not affected by partial volume effects.

### DEXA Measurement

All DEXA measurements were performed by the same investigator (Y. C) using the Norland pDEXA Sabre (Fort Atkinson, WI, USA) equipped with Sabre Research software (v3.6). The interassay coefficient of variation (CV) for BMD and BMC was 1.7%. The scanner was calibrated daily to a dual-material standard according to the manufacturer's recommendations, and the scanner performance was controlled by the quality assurance protocol of our laboratory. The right femurs were scanned using DEXA to determine BMC and BMD. *Ex vivo *measurements of the right distal femur were performed on excised bones positioned onto a 3-mm-thick cotton piece on the bottom (thickness 1 mm) of a 10-cm diameter culture dish at a constant location on the scan table, and measured by DEXA using a special collimator (0.8 mm diameter); the scan length was 5 cm, the scan width 2 cm and the scan speed 10 mm/s with a resolution of 0.2 mm × 0.2 mm [16-18]. The deltoid tuberosity was faced upward to avoid an irregular projecting shape; the starting point of the scan was above the distal condyle of the femur and the end point was proximal to the femoral end so that the scanner arm moved along the long axis of the femoral shaft allowing evaluation of femur length. The baseline point was located on the cotton piece [16,19].

### Liver Histological Examination

Liver specimens were fixed in 10% buffered neutral paraformaldehyde solution, processed and embedded in paraffin. Thin paraffin sections (5-μm thick) were stained by hematoxylin and eosin (H & E). The numbers of mononuclear cells were determined/10 HPF.

### Bone Histomorphometric Analysis

Left tibiae were decalcified in 5% formic acid (in distilled water) solution for 1 week, dehydrated with methanol, and embedded in paraffin. The paraffin sections were deparaffinized and stained (H & E). Sections with the widest marrow cavity near the growth plate of the metaphysis of tibiae were selected for further histological processing and histomorphometric measurements.

Histomorphometrical measurements were made using an Optiphot 2 microscope connected to a RGB camera and a personal computer (software: Lucia G 4.51, Laboratory Imaging), with final magnifications of 30× and 400×. The number of osteoclasts (Oc.N) was determined/10 HPF.

### BALP Enzyme Assay

Rat bone alkaline phosphatase (BALP) enzyme-linked immunosorbent assay (ELISA) kit was provided by Cusabio Biotech Co., LTD. (Wuhan, China). Rat BALP was also measured using ELISA from R & D Systems (Minneapolis, MN, USA).

### TRAP Enzyme Assay

Rat TRAP-5b EIA Kit was obtained from KAMIYA BIOMEDICAL Company (Seattle, WA, USA). Rat TRAP-5b was also measured by ELISA (R & D Systems, Minneapolis, MN, USA).

### Plasma Peroxide Assay

The plasma malondialdehyde (MDA) levels were determined according to the method of Draper and Hadley (1990) [20], based on the reaction of MDA with thiobarbituric acid. Measurement was conducted using the lipid peroxidation assay kit (Calbiochem). The absorbance at 586 nm was measured using an ELISA microplate reader.

### Plasma Nitrate Assay

Plasma nitrate levels were measured according to the method of Bories and Bories (1995) [21]. Total serum nitric oxide (NO) was calculated based on the enzymatic conversion of nitrate to nitrite by nitrate reductase, using a commercial kit (Total Nitric Oxide and Nitrate/Nitrite Parameter Assay Kit, R&D SYSTEMS, Minneapolis, MN, USA).

### Biochemical Analysis of Serum Parameters

Serum content of calcium, inorganic phosphorus (IP), ALP, triiodothyronine (T_3_), thyroxine (T_4_), osteocalcin, estradiol, intact PHT and calcitonin were determined using standard laboratory techniques. Serum levels of free T_4_, free T_3_, intact PTH, and estradiol were measured with free T_3_, free T_4_, Elecys PTH, and Estradiol α kits (Roche, Germany), respectively, using Modular Analytics E170 (Roche, Germany) in the electrochemiluminescence immunoassay method. Serum calcium and IP were measured with related kits (Roche, Germany) using Modular Analytics PE (Roche, Germany) in the colorimetric and phosphomolybdate & ultraviolet spectrophotometric methods, respectively. Serum ALP activity was measured with ALP kit (Roche, Germany) using Modular Analytics PE (Roche, Germany) with colorimetry with PNPP. Calcitonin was measured with Liaison calcitonin α-Gen kit (Diasorin, USA) by the chemiluminescent immunoassay method.

### Statistical Analysis

Data are expressed as means ± SD. Statistical significance for data was determined using one-way analysis of variance (ANOVA) with post-hoc test, and significance was calculated by LSD (least significant difference) multiple range-test to find inter-group significance. The level of significance was accepted as *p <*0.05.

## Results

### Preparation of SM extracts

In the pure components of SM, tanshinone I, tanshinone IIA, tanshinone IIB, cryptotanshinone, tanshindiol C, 15,16-dihydrotanshinone I, isotanshinone I, isotanshinone II and other tanshinones are included [4]. Among the tanshinone compounds, tanshinone IIA and cryptotanshinone were selected as active and quality control compounds in this study. Calibration curves of the two compounds were constructed by measuring different concentrations. Good linearity was observed for tanshinone IIA (r^2 ^= 0.9911) and cryptotanshinone (r^2 ^= 0.9921). The regression equations for t`anshinone IIA and cryptotanshinone were y = 59467x + 296829 and y = 62354x - 109248, respectively (data not shown). The typical HPLC-UV profiles are illustrated in Additional file 1. The HPLC condition has been also described in Additional file 2. Good separation was achieved within 25 min. The retention times for cryptotanshinone and Tanshinone IIA were 14.8 and 21.6 min. The content of tanshinone IIA and cryptotanshinone in *Salvia Miltiorrhiza *was determined from the corresponding regression equation. Tanshinone IIA content was 106.56 μg/10 mg of SM extract (1.07%) whereas cryptotanshinone content was 109.655 μg/10 mg of *SM *extract (1.10%).

### Body Weight Changes

As time passed from 2 to 8 weeks after OVX, the average body weight growth in the OVX groups was significantly greater than that in the Sham group (*p *< 0.05~0.001), but administration of SM did not affect the body weight growth pattern (Additional file 3).

### BMD and BMC

**Figure 1:**
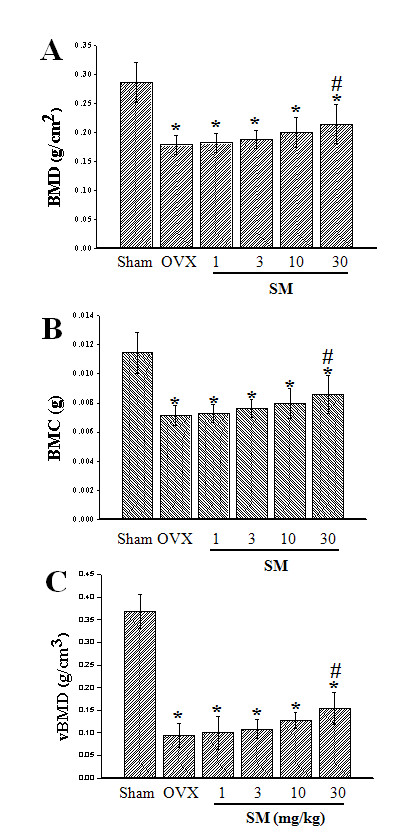
**The Quantification of BMD and BMC in SM-treated rats**. aBMD (A) and aBMC (B) of right distal femur *ex vivo *measured by DEXA, and vBMD (C) of right proximal tibia *ex vivo *measured μ-CT. OVX significantly decreased aBMD,by aBMC and vBMD, respectively. Although SM treatment tended to have a dose-dependent preventive effect, only treatment with 30 mg/kg body weight/day of SM significantly prevented BMD and BMC decrease induced by OVX. Note: *p < 0.001 vs Sham group; ^#^*p *< 0.05 vs OVX group

**Figure 2:**
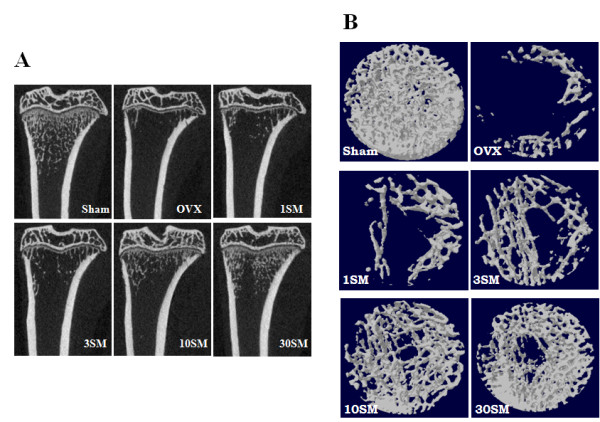
**The image analysis of rat medial-proximal tibia in SM-treated rats**. A, Coronal images of rat medial-proximal tibia were shown by μ-CT. B, 3D images taken by μ-CT.

**Figure 3:**
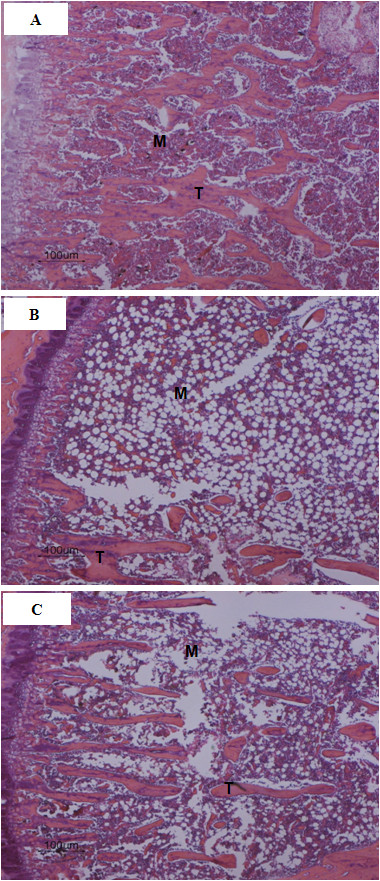
**Bone histochemical section images of proximal metaphysis of tibia**. A, Sham group: trabecular bone (T) and bone marrow spaces (M). B, OVX group: significantly increased bone marrow space, decreased trabecular network. C, 30 mg/kg of SM treatment significantly attenuated trabecular network loss compared with Sham rats. (H & E, ×100).

In DEXA *ex vivo *measurement, the aBMD and aBMC of right distal femora were significantly decreased by 38%, respectively, by OVX (*p *< 0.001). SM administration provided some degree of safety in a dose-dependent manner, but only high-dosage SM (30 mg/kg body weight/day) treatment significantly prevented aBMD and aBMC reduction by 33%, respectively (*p *< 0.05) (Figure 1A, B). In μ-CT *ex vivo *measurement, the vBMD of proximal tibiae was significantly reduced by 74% (*p *< 0.001), and SM treatment resulted in the same pattern as in DEXA measurement, i.e., the vBMD decrease was prevented by 22% only in 30SM rats (*p *< 0.05) (Figure 1C). This study showed the coronal images of rat medial-proximal tibia by μ-CT (Figure 2A) and 3D images μ-CT (Figure 2B) with the taken by SM dose-dependent prevention about bone loss in OVX rats.

### μ-CT Evaluation

To examine the effect of SM on BMD, coronal image of proximal-medial tibia was taken ex vivo by μ-CT. A. Additional file 4 showed setting conditions for the μ-CT. Table 1 showed that OVX induced significant changes in all trabecular microstructural parameters in the proximal tibial metaphysis measured by μ-CT. Compared with Sham rats, OVX significantly reduced bone volume fraction (BV/TV), by 87% (*p *< 0.001), trabecular thickness (Tb.Th) by 14% (*p *< 0.001), trabecular number (Tb.N) by 85% (*p *< 0.001) and connectivity density (Conn.D) by 91% (*p *< 0.001), and increased trabecular separation (Tb.Sp) by 320% (*p *< 0.001). Other microstructural parameters such as SMI and trabecular bone pattern (Tb.Pf) were also significantly different (*p *< 0.001). SM treatment also showed some tendency for dose-dependent safety effects but only the maximum SM treatment of 30 mg/kg had a significant preventive effect, attenuating reduction of BV/TV by 24% (*p *< 0.05), Tb.Th by 65% (*p *< 0.05), Tb.N by 23% (*p *< 0.05) and Conn.D by 12% (*p *< 0.05), while preventing increase of Tb.Sp by 43% (*p *< 0.05), SMI by 30% (*p *< 0.05) and Tb.Pf by 28% (*p *< 0.05). Ct. Ar and Ct. Th measured by μ-CT were also summarized in the Table 1. OVX did not affect the cortical area and thickness of tibial diaphysis.

**Table 1:** Trabecular microstructural properties of the right tibial metaphysis and cortical geometric properties evaluated *ex vivo *using micro-CT.

Parameters	Sham n = 10	OVX n = 10	SM1 n = 7	SM3 n = 8	SM10 n = 10	SM30 n = 9
**BV/TV (%)**	30.06 ± 4.57	3.90 ± 1.61 *	4.56 ± 2.44 *	4.92 ± 1.84 *	6.22 ± 1.78 *	8.05 ± 3.32 *, ^#^
**Tb.Th (μm)**	112.76 ± 7.95	96.51 ± 4.99 *	101.03 ± 6.58 *	100.06 ± 7.42 *	99.53 ± 5.92 *	107.03 ± 5.02 *, ^#^
**Tb.N (1/mm)**	2.67 ± 0.40	0.40 ± 0.16 *	0.45 ± 0.23 *	0.50 ± 0.19 *	0.63 ± 0.19 *	0.93 ± 0.28 *, ^#^
**Tb.Sp (mm)**	0.25 ± 0.08	1.05 ± 0.13 *	1.05 ± 0.26 *	1.03 ± 0.20 *	0.93 ± 0.21 *	0.71 ± 0.10 *, ^#^
**Conn.D (1/mm^3^)**	97.53 ± 12.90	8.95 ± 3.67 *	9.24 ± 4.90 *	10.59 ± 3.22 *	14.24 ± 6.62 *	19.37 ± 6.34 *, ^#^
**Tb.Pf (1/mm)**	4.25 ± 2.29	20.35 ± 2.98 *	19.50 ± 2.26 *	18.98 ± 1.89 *	18.48 ± 2.62 *	15.79 ± 3.05 *, ^#^
**SMI (1)**	1.46 ± 0.25	2.69 ± 0.10 *	2.64 ± 0.12 *	2.61 ± 0.14 *	2.59 ± 0.16 *	2.32 ± 0.26 *, ^#^
**Ct.Ar (mm^2^)**	5.38 ± 0.22	5.41 ± 0.45	5.98 ± 0.34	5.67 ± 0.43	5.58 ± 0.34	5.29 ± 0.31
**Ct.Th (mm)**	29.65 ± 0.13	29.63 ± 0.09	29.65 ± 0.07	29.54 ± 0.10	29.62 ± 0.06	29.66 ± 0.08

**Note**: Results are presented as the means ± SD. *p < 0.001 vs. Sham group; ^#^*p *< 0.05 vs. OVX group. BV/TV, bone volume fraction; Conn.D, connectivity density; SMI, structure model index; Tb.Th, trabecular thickness; Tb.N, trabecular number; Tb.Sp, trabecular separation; Tb.Pf, Trabecular bone pattern factor; Ct.Ar, cortical area; Ct.Th, cortical thickness. Rat groups include sham-operated (Sham), OVX-control (OVX), and 1, 3, 10 and 30 mg/kg SM treated (1SM, 3SM, 10SM AND 30SM) ovariectomized groups for a total of 6 groups (N = 7~10/each group).

### Bone Histomorphometric Parameters

As shown in Table 2 and Figure 3, the histomorphometric parameters were analogous to the μ-CT observations of trabecular morphology: OVX significantly reduced BV/TV by 82% (*p *< 0.001), Tb.Th by 58% (*p *< 0.001), Tb.N by 64% (*p *< 0.001), and increased Tb.Sp by 604% (*p *< 0.001). SM treatment also tended to have a dose-dependent preventive effect at the experimental dosages, but only treatment with the maximum of 30 mg/kg body weight/kg of SM showed significance, attenuating reduction of BV/TV by 19% (*p *< 0.05), Tb.Th by 57% (*p *< 0.05), and Tb.N by 65% (*p *< 0.05), while preventing the increase of Tb.Sp by 69% (*p *< 0.05). OVX also induced a significant increase in Oc.N (*p *< 0.001 vs. Sham), and SM treatment attenuated the Oc.N increase only in the 30SM group (*p *< 0.05 vs. OVX).

**Table 2:** Histomorphometric parameters of trabecular bone from metaphysis of tibiae evaluated by histochemical analysis.

Parameters	Sham n = 7	OVX n = 7	1SM n = 7	3SM n = 7	10SM n = 7	30SM n = 7
**BV/TV (%)**	55.44 ± 7.10	10.24 ± 3.68 *	11.51 ± 2.27	11.61 ± 2.27	15.19 ± 1.61	18.70 ± 1.38 ^#^
**Tb.Th (mcm)**	35.14 ± 5.17	14.86 ± 5.54 *	15.72 ± 3.33	16.37 ± 3.03	21.32 ± 3.71	26.37 ± 2.49 ^#^
**Tb.N (1/mm)**	15.87 ± 1.59	5.73 ± 2.03 *	6.06 ± 1.99	6.39 ± 2.00	8.88 ± 2.61	12.35 ± 2.67 ^#^
**Tb.Sp (mcm)**	28.38 ± 5.80	199.70 ± 3.48 *	180.27 ± 3.46	176.26 ± 2.18	98.75 ± 7.21	81.67 ± 6.38 ^#^
**Oc.N/10 HPF**	5 ± 1.41	17 ± 2.40 *	16 ± 2.39	17 ± 2.20	13 ± 2.26	10 ± 1.41 ^#^

**Note**: Results are presented as the means ± SD. *p < 0.001 vs. Sham group; ^#^*p *< 0.05 vs. OVX group. BV/TV, bone volume fraction; Tb.Th, trabecular thickness; Tb.N, trabecular number; Tb.Sp, trabecular separation; Oc.N, osteoclast number. Rat groups as defined above (N = 7/each group).

### Liver Histomorphometry

**Figure 4:**
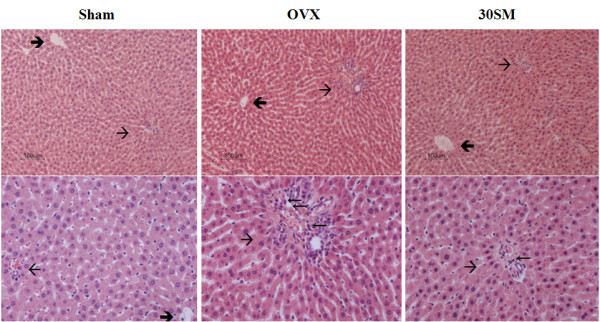
**Liver histochemical section images**. Plates of liver parenchymal cells and sinusoids among the laminae of hepatic cells radiating from the central vein (→) and radiating towards the portal area (→). Upper (H & E, ×100) and Lower (H & E, ×200).

As shown in Figure 4 and Table 3, OVX aggravated mononuclear cellular infiltration in the portal area of the liver (*p *< 0.001 vs. Sham rats) and SM treatment significantly ameliorated mononuclear cellular infiltration only at 30 mg/kg body weight/day (*p *< 0.05 vs. OVX rats).

**Table 3:** The number of infiltrated mononuclear cells in the portal area of liver/10 HPF (×200).

Parameters	Sham n = 7	OVX n = 7	1SM n = 7	3SM n = 7	10SM n = 7	30SM n = 7
**Infiltrated Mononuclear cellular NO/10 HPF**	1.57 ± 1.27	12.00 ± 2.40 *	12.00 ± 2.39	11.50 ± 2.20	10.38 ± 2.26	8.50 ± 2.34 ^#^

**Note**: Results are presented as the means ± SD. *p < 0.001 vs. Sham group; ^#^*p *< 0.05 vs. OVX group. Rat groups as defined above (N = 7/each group).

### Quantification of Serum bone turnover markers

**Figure 5:**
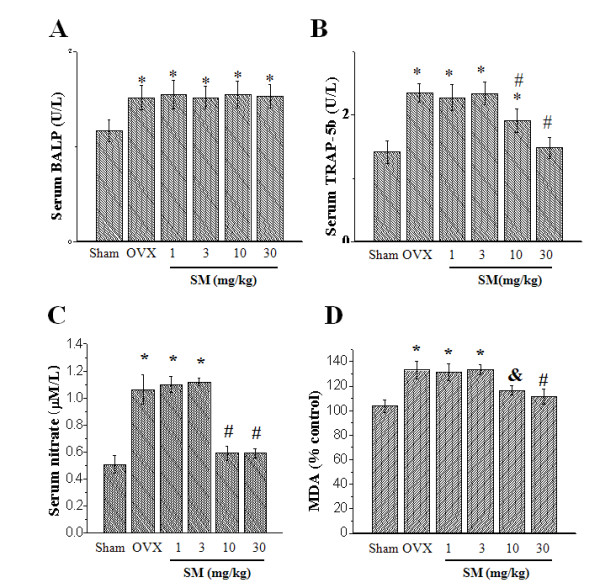
**Serum bone turnover marker levels**. Serum BALP activity (A), seru m TRAP-5b activity (B), Serum MDA levels (C) and serum nitrate levels (D) were measured as described in Materials and Methods. Note: (A) to (B) *p < 0.05 vs Sham rats; ^#^*p *< 0.05 vs OVX rats. (C) *p < 0.001 vs Sham group; ^#^*p *< 0.001 vs OVX group. (D) *p < 0.001 vs Sham rats; ^#^*p *< 0.001 vs OVX rats; ^&^*p *< 0.01 vs OVX rats.

As shown in Figure 5A, serum BALP as a bone formation marker [22] was significantly increased in OVX rats (*p *< 0.05), while drug treatment did not affect the increase.

TRAP 5b in serum is proposed to be a marker for osteoclasts [23]. As shown in Figure 5B, serum TRAP 5b was significantly increased in OVX rats compared with Sham group (*p *< 0.05) but was significantly attenuated in 30SM group (*p *< 0.05), consistent with exchange in osteoclast number measured by histological assessment (Table 2) and indicating increased bone resorption. In order to understand the mechanism of SM on bone resportion parameter, malondialdehyde (MDA) and nitric oxide (NO) were measured. OVX significantly increased serum MDA levels (*p *< 0.001 vs. Sham rats), meaning the induction of lipid peroxydation in OVX rats (Figure 5C). SM treatment, especially at the two groups, 10 and 30SM, significantly attenuated the MDA increase induced by OVX (*p *< 0.001 and *p *< 0.01, respectively). Figure 5D showed that OVX significantly increased total serum nitrate, metabolite of NO (*p *< 0.001 vs. Sham rats), and in 10SM and 30SM rats, SM treatment significantly prevented the nitrate increase induced by OVX (*p *< 0.001 vs. Sham rats).

### Serum Biochemical Levels

Serum calcitonin and intact PTH levels were not significantly different among experimental groups (data not shown). As shown in Table 4, serum calcium and IP levels and free T_3 _were not significantly different among experimental groups, while OVX significantly decreased estradiol (*p *< 0.01) but the SM did not affect the decrease of estradiol. Free T4 was significantly increased in OVX rats (*p *< 0.001) and the increase was significantly attenuated in 30SM rats (*p *< 0.001). OVX significantly increased serum osteocalcin and ALP activity (*p *< 0.001 and 0.05, respectively) and SM treatment did not affect the increase.

**Table 4:** Serum biochemical parameters.

Parameters	Sham n = 10	OVX n = 10	SM1 n = 7	SM3 n = 8	SM10 n = 10	SM30 n = 9
**ALP (U/L)**	32.57 ± 8.28	51.29 ± 7.78 ^&^	47.71 ± 5.38	51.57 ± 3.74	51.14 ± 8.80	47.57 ± 7.00
**Estradiol (pg/mL)**	16.09 ± 3.38	9.29 ± 2.51 ^#^	10.27 ± 3.18	8.10 ± 3.03	9.07 ± 2.60	10.10 ± 3.23
**Free T3 (pg/mL)**	3.84 ± 0.50	3.83 ± 0.31	3.97 ± 0.31	3.91 ± 0.23	3.79 ± 0.41	3.74 ± 0.23
**Free T4 (ng/dL)**	1.90 ± 0.47	3.04 ± 0.25 *	3.07 ± 0.30	2.89 ± 0.31	2.73 ± 0.23	2.00 ± 0.51 ^%^
**Osteocalcin (ng/mL)**	2.40 ± 0.28	3.66 ± 0.48 *	3.40 ± 0.57	3.63 ± 0.44	3.44 ± 0.43	3.47 ± 0.42
**Calcium (mg/dL)**	9.30 ± 0.41	9.46 ± 0.29	9.63 ± 0.21	9.78 ± 0.46	9.40 ± 0.27	9.70 ± 0.51
**IP (mg/dL)**	6.21 ± 0.80	6.24 ± 0.65	6.60 ± 0.57	6.50 ± 0.72	6.17 ± 0.53	6.49 ± 0.79

Note: T3, triiodothyronine; T4, thyroxine, IP, inorganic phosphorus. Results are presented as the means ± SD. *p < 0.001 vs. Sham group; ^#^*p*< 0.01 vs. Sham group; ^&^*p *< 0.05 vs. Sham group; ^%^*p *< 0.001 vs. OVX group. Rat groups as defined above (N = 6/each group).

## Discussion

OVX induced significant trabecular bone loss due to estrogen deficiency and subsequent increased bone turnover. SM at 30 mg/kg body weight/day dosage significantly attenuated trabecular bone loss and BMD decrease induced by OVX. SM can contribute to bone balance probably through preventing an increase in osteoclast number by decreasing osteoclast maturation.

SM is a potential anti-osteoporotic natural product. For several decades, SM has been widely used for the treatment of various microcirculatory disturbance-related diseases, such as cardiovascular disease, cerebrovascular disease, liver dysfunction, renal deficiency and diabetic vascular complications [4]. SM extract is also reported to increase bone formation through the combined actions of increased angiogenesis, increased osteoblastic activity and decreased osteoclastic activity [10]. In the current study, treatment with 30 mg/kg of ethanol extracts of SM significantly attenuated the dramatic decrease in BMD and deterioration in trabecular bone architecture (Table 1 &2).

SM treatment also significantly prevented increases in serum nitrate and peroxide levels and ameliorated the increase in mononuclear cellular infiltration in the portal area of the liver (Table 3). In the current study, histological examination of the liver of the SM treated rats showed the regulatory effect of mononuclear cellular infiltration (Figure 4). Previous studies have showed that OVX condition induces liver inflammation [24,25]. The estrogen-induced prevention effect against bone loss may involve suppression of inflammatory cytokines such as IL 1, IL-6 or TNF-α, which in turn activate inducible nitric oxide synthase (iNOS). Nitric oxide (NO) is derived from the iNOS pathway potentiates the inflammatory cytokine-associated bone loss [25]. These studies give a possible explanation for the detected significant increase in the plasma nitrates level present in the OVX-rats in our study. Malondialdehyde (MDA) was also significantly increased in the OVX rats indicating increased oxidative stress. In the current study, SM treatment regulated the production of NO and MDA, which are related with bone resorption. It has been demonstrated that free radicals intervene in bone resorption, promoting osteoclastic differentiation [26]. Considering that enhanced osteoclastic activity in OVX rats has been suggested to be responsible for increased ROS [27], the regulatory effect of SM on NO and MDA could be one of the anti-osteoporotic mechanisms of the natural product.

In this study, SM treatment also significantly attenuated the increase in bone osteoclast number and serum TRAP-5b but did not affect the increase in serum BALP and ALP or in osteocalcin and estradiol induced by OVX. Generally, in subjects with normal liver function, serum ALP is similar to BALP and reflects osteoblast function [28]. Together with osteocalcin, they are markers of bone formation, while TRAP-5b is a bone resorption marker [22]. In the serum biochemical assessment, OVX did not affect serum calcium and IP levels or PTH and calcitonin activity, but significantly increased free T_4 _activity compared with Sham rats (Table 4). Free T_4 _activity was significantly reduced in 30SM rats compared with OVX rats (also shown in Table 4). Thyroid hormones play an important role in bone remodeling [29]. Histomorphometric studies have shown that thyroid hormones stimulate osteoblastic and osteoclastic activities in cortical and trabecular bone [30]. Thyrotoxicosis is associated with increased bone turnover, which can lead to a resorption rate that exceeds the formation rate, thus resulting in bone loss [31]. Considering that an increased rate of bone turnover was observed in subjects loaded with suppressive doses of T_4_, the inhibition of the increase of T_4 _levels by SM further suggests that SM has a regulatory effect on bone turnover. Increases in bone turnover have been reported in the perimenopausal period in humans probably due to estrogen deficiency [32]. Consistently, estradiol decrease was observed in OVX rats (Table 4). The reduced estradiol was not recovered by SM treatment. But with the data about estrogen, we could not determine whether SM has hormone-like effect or not. Although we did not clarify the characteristics of SM about hormone-like effect, we are suggesting that SM prevents trabecular bone loss by modulating osteoclast activity including decreasing osteoclast number/by decreasing osteoclast maturation, resulting in the regulation of bone turnover rate rather than by deceasing estrogen level.

The pharmacokinetics studies of these active components of SM in animals showed that they are absorbed orally and randomized clinical trials and clinical experiences indicate that the SM products are safe with a low side-effect profile [5]. Therefore, SM is a promising osteoporosis therapeutic agent candidate, although the specific mechanism of the anti-osteoporotic effect of SM needs to be clarified.

## Conclusions

The preventive effect of SM against osteoporosis was presumably due to its anti-oxidative stress partly via modulation of osteoclast maturation and number. In current study, SM has been suggested to be a promising osteoporosis therapeutic natural product.

## Abbreviations

ALP: alkaline phosphatase; aBMD: bone mineral density measured by DEXA; BMD: bone mineral density measured by micro-CT; aBMC: bone mineral content measured by DEXA; BV/TV: bone volume fraction; BALP: bone-specific alkaline phosphatase; BTM: bone turnover marker; DEXA: dual-energy X-ray absorptiometry; ELISA: enzyme-linked immunosorbent assay; HPLC: high performance liquid chromatography; IL: interleukin; iNOS: inducible nitric oxide synthase; IP: inorganic phosphorus; M-CSF: macrophage colony stimulating factor; MDA: malondialdehyde; μ-CT: microcomputerized tomography; NO: nitric oxide; NTX: amino-terminal region of Telopeptides of type I collagen; Oc.N: osteoclast number; OVX: ovariectomy or ovariectomized; PTH: parathyroid hormone; ROS: reactive oxygen species; SD: standard deviation; SMI: structure model index; T3: triiodothyronine; T4: thyroxine; Tb.N: trabecular number; Tb.Pf: trabecular bone pattern factor; Tb.Sp: trabecular separation; Tb.Th: trabecular thickness; TNF-α: tumor necrosis factor α; TRAP: tartrate-resistant acid phosphatase; TRAP-5b: type 5 tartrate resistant acid phosphatase band

## Competing interests

We declare that we have no competing interests. We also declare that we have no financial and non-financial competing interests.

## Authors' contributions

YC mainly performed the animal experiment, analyzed the data and wrote a draft. AM did quantitative analysis of danshen. SC partially wrote a draft. BB, GL, BL and DK supported the animal experiment, especially for feeding and establishing osteoporosis model. HK and HC supervised the project and wrote the final paper. All authors read and approved the final manuscript.

## Pre-publication history

The pre-publication history for this paper can be accessed here:

http://www.biomedcentral.com/1472-6882/11/120/prepub

## Supplementary Material

Additional file 1 **Chromatogram of constituents from SM extracts by HPLC analysis**. The chromatogram shows peaks about Tanshinone IIA and cryptotanshinone. The retention time for cryptotanshinone and tanshinone IIA was 14.8 and 21.6 min. The content of tanshinone IIA and cryptotanshinone in *Salvia Miltiorrhiza *was 106.56 μg/10 mg (1.07%) and 109.655 μg/10 mg (1.10%). Click here for file

Additional file 2 **HPLC analysis methods for measurement of the standards of Tanshinone IIA and Cryptotanshinone**. The Additional file shows HPLC analysis method for the standard chemicals "Tanshinone IIA and Cryptotanshinone". Click here for file

Additional file 3 **Changes in body weight growth in rats**. The additional file shows the rat's body weights from the second week after OVX (including OVX and all drug administration groups). Click here for file

Additional file 4 **Coronal image of proximal-medial tibia taken *ex vivo *by μ-CT**. The additional file shows conditions for μ-CT (references Set distal growth plate as reference level and 8 mm distal from distal growth plate as cortical area analysis cut level and the related transaxial image of tibial diaphysis). Click here for file
