# Supplementary material for: Characterization of Salvia Miltiorrhiza ethanol extract as an anti-osteoporotic agent
Source: BMC Complement Altern Med. 2011 Nov 28;11:120. doi: 10.1186/1472-6882-11-120 (PMC3298536; doi:10.1186/1472-6882-11-120)
Supplement: Additional file 1 — Chromatogram of constituents from SM extracts by HPLC analysis. The chromatogram shows peaks about Tanshinone IIA and cryptotanshinone. The retention time for cryptotanshinone and tanshinone IIA was 14.8 and 21.6 min. The content of tanshinone IIA and cryptotanshinone in Salvia Miltiorrhiza was 106.56 μg/10 mg (1.07%) and 109.655 μg/10 mg (1.10%). [file 1472-6882-11-120-S1.PPTX]

## Slide 1
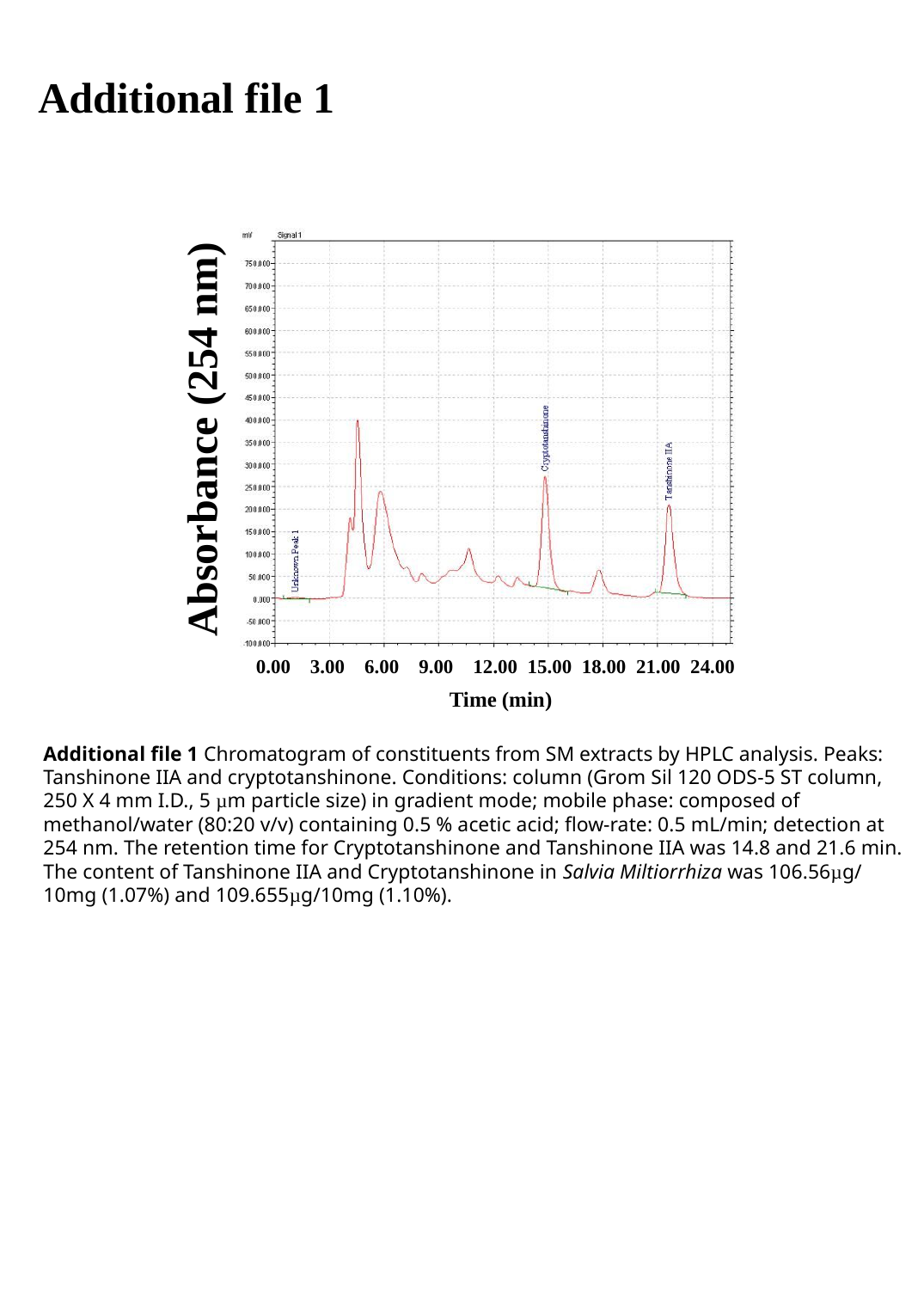

Additional file 1
Absorbance (254 nm)
0.00 3.00 6.00 9.00 12.00 15.00 18.00 21.00 24.00
Time (min)
Additional file 1 Chromatogram of constituents from SM extracts by HPLC analysis. Peaks: Tanshinone IIA and cryptotanshinone. Conditions: column (Grom Sil 120 ODS-5 ST column, 250 X 4 mm I.D., 5 µm particle size) in gradient mode; mobile phase: composed of methanol/water (80:20 v/v) containing 0.5 % acetic acid; flow-rate: 0.5 mL/min; detection at 254 nm. The retention time for Cryptotanshinone and Tanshinone IIA was 14.8 and 21.6 min. The content of Tanshinone IIA and Cryptotanshinone in Salvia Miltiorrhiza was 106.56µg/10mg (1.07%) and 109.655µg/10mg (1.10%).
