# Supplementary material for: Characterization of Salvia Miltiorrhiza ethanol extract as an anti-osteoporotic agent
Source: BMC Complement Altern Med. 2011 Nov 28;11:120. doi: 10.1186/1472-6882-11-120 (PMC3298536; doi:10.1186/1472-6882-11-120)
Supplement: Additional file 2 — HPLC analysis methods for measurement of the standards of Tanshinone IIA and Cryptotanshinone. The Additional file shows HPLC analysis method for the standard chemicals "Tanshinone IIA and Cryptotanshinone". [file 1472-6882-11-120-S2.DOCX]

***Additional file 2***

*HPLC analysis*

The high performance liquid chromatography equipment consisted of auto sampler (Model S5200), solvent delivery system (Model S2100) and UV-Vis detector (Model S3210S) from Sykam (Munich, Germany). Tanshinone IIA and Cryptotanshinone were separated on Grom Sil 120 ODS-5 ST column, 250 X 4 mm I.D., 5-µm particle size. The mobile phase was composed of methanol/water (80:20 v/v) containing 0.5% acetic acid. This mobile phase was filtered through a 0.45-µm membrane filter (Osmonics), then deaerated ultrasonically prior to use. The flow rate was 0.5 mL/min. The UV wavelength was 254 nm. The column temperature was 50°C. The chromatographic peaks of the analytes were confirmed by comparing their retention times and UV spectra with those of the reference standards. Quantification was carried out by integration of the peak using the external standard method.

The standards of Tanshinone IIA and Cryptotanshinone were dissolved in methanol to a final concentration of 1 mg/mL and stored at 4°C. The stock solutions were then diluted with methanol to obtain a series of solutions with different concentrations.
