# Supplementary material for: Characterization of Salvia Miltiorrhiza ethanol extract as an anti-osteoporotic agent
Source: BMC Complement Altern Med. 2011 Nov 28;11:120. doi: 10.1186/1472-6882-11-120 (PMC3298536; doi:10.1186/1472-6882-11-120)
Supplement: Additional file 3 — Changes in body weight growth in rats. The additional file shows the rat's body weights from the second week after OVX (including OVX and all drug administration groups). [file 1472-6882-11-120-S3.PPTX]

## Slide 1
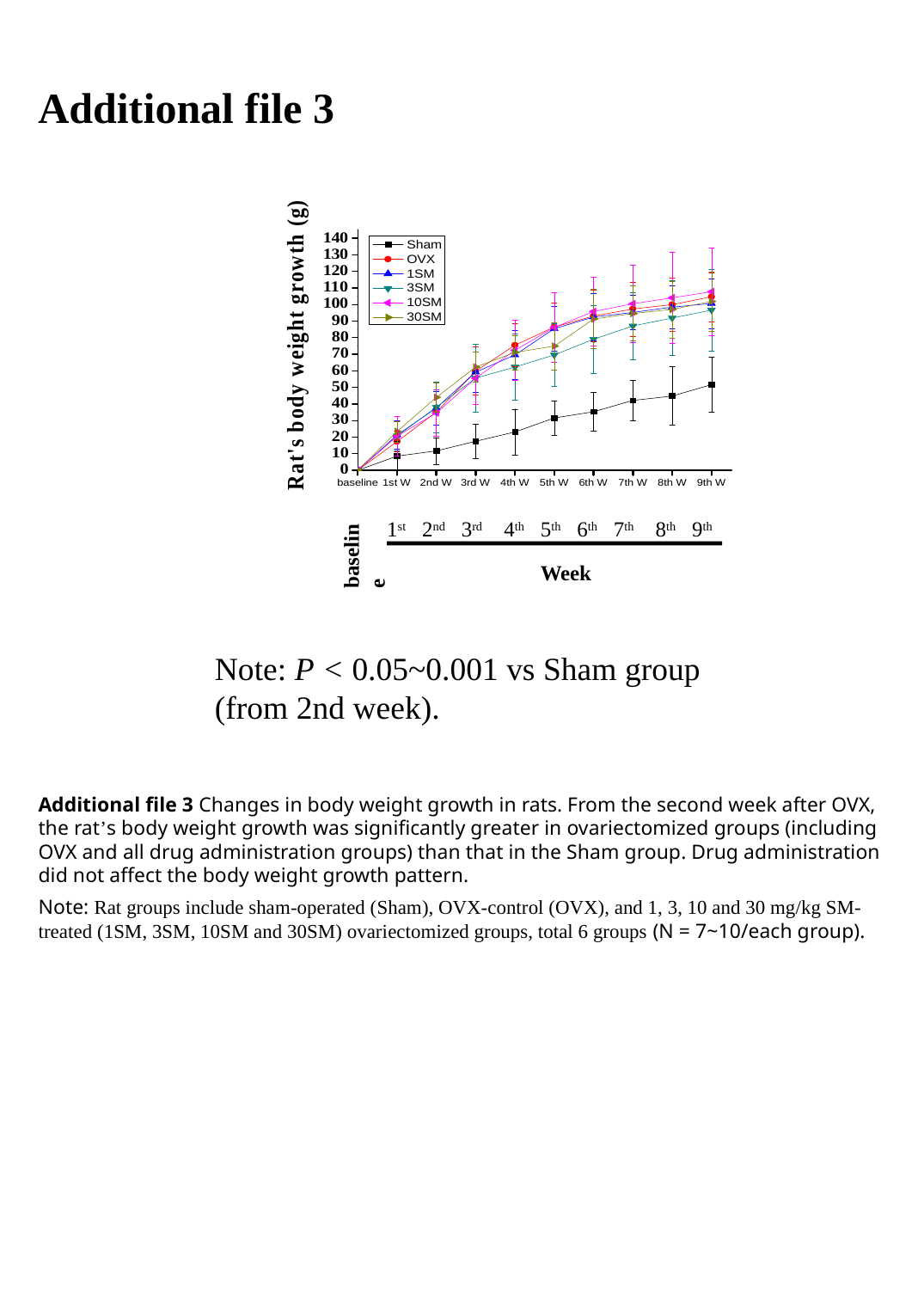

Additional file 3
 1st 2nd 3rd 4th 5th 6th 7th 8th 9th
baseline
Week
Note: P < 0.05~0.001 vs Sham group (from 2nd week).
Additional file 3 Changes in body weight growth in rats. From the second week after OVX, the rat’s body weight growth was significantly greater in ovariectomized groups (including OVX and all drug administration groups) than that in the Sham group. Drug administration did not affect the body weight growth pattern.
Note: Rat groups include sham-operated (Sham), OVX-control (OVX), and 1, 3, 10 and 30 mg/kg SM-treated (1SM, 3SM, 10SM and 30SM) ovariectomized groups, total 6 groups (N = 7~10/each group).
