# Supplementary material for: Characterization of Salvia Miltiorrhiza ethanol extract as an anti-osteoporotic agent
Source: BMC Complement Altern Med. 2011 Nov 28;11:120. doi: 10.1186/1472-6882-11-120 (PMC3298536; doi:10.1186/1472-6882-11-120)
Supplement: Additional file 4 — Coronal image of proximal-medial tibia taken ex vivo by μ-CT. The additional file shows conditions for μ-CT (references Set distal growth plate as reference level and 8 mm distal from distal growth plate as cortical area analysis cut level and the related transaxial image of tibial diaphysis). [file 1472-6882-11-120-S4.PPTX]

## Slide 1
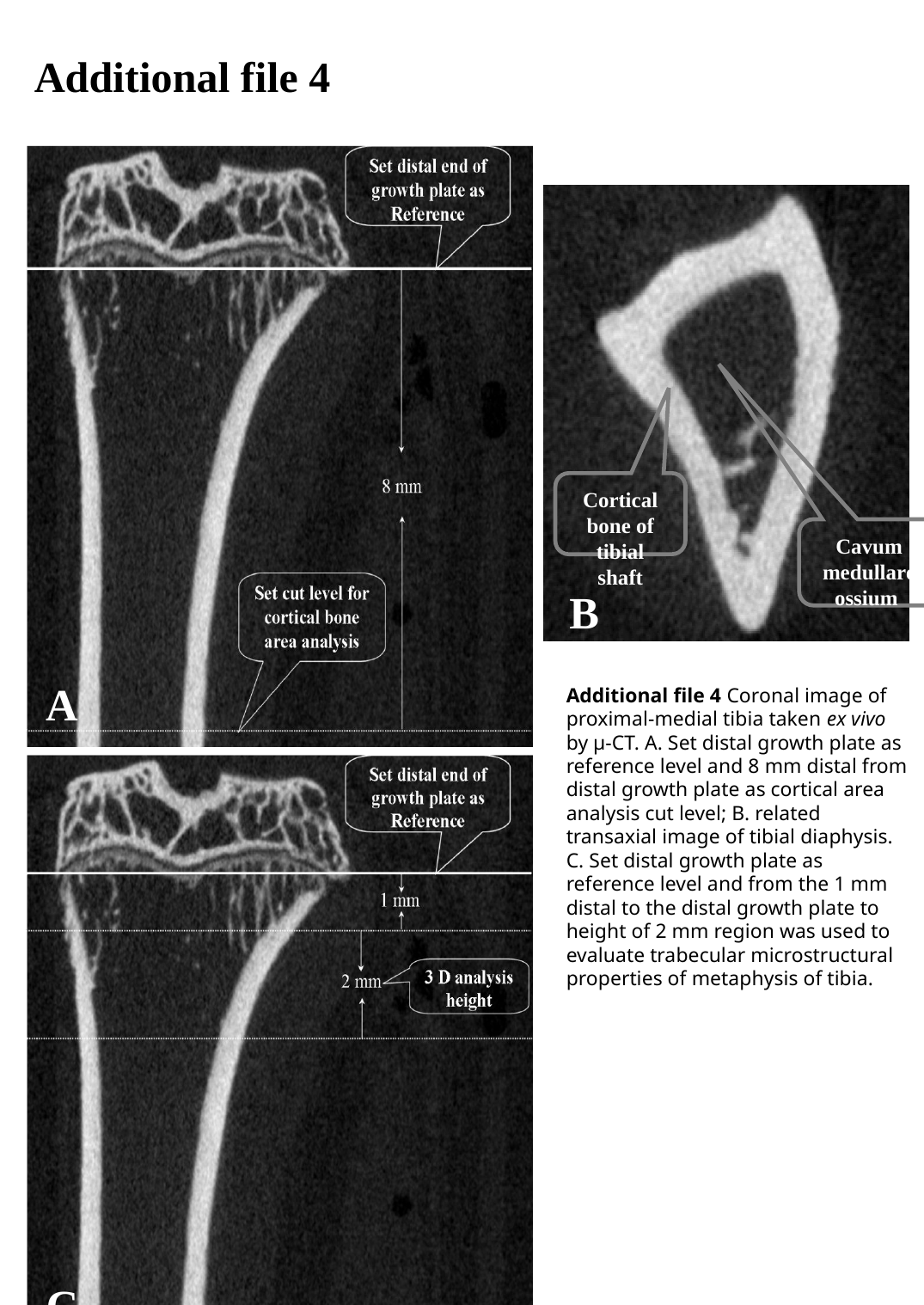

Additional file 4
A
Cortical bone of tibial shaft
Cavum medullare ossium
B
Additional file 4 Coronal image of proximal-medial tibia taken ex vivo by μ-CT. A. Set distal growth plate as reference level and 8 mm distal from distal growth plate as cortical area analysis cut level; B. related transaxial image of tibial diaphysis. C. Set distal growth plate as reference level and from the 1 mm distal to the distal growth plate to height of 2 mm region was used to evaluate trabecular microstructural properties of metaphysis of tibia.
C
